# Application of Organic Amine Modified Natural Zeolite in Filling Natural Rubber

**DOI:** 10.3390/nano12162784

**Published:** 2022-08-14

**Authors:** Xiaolong Chen, Taozhong Zhang, Pengliang Sun, Fujia Yu, Bin Li, Linan Dun

**Affiliations:** 1 College of Resources and Civil Engineering, Northeastern University, Shenyang 110004, China; 2 School of Chemical Science and Technology, Yunnan University, Kunming 650091, China; 3 College of Materials Science and Engineering, Northeastern University, Shenyang 110004, China

**Keywords:** zeolite, organic amine, surface modification, natural rubber, composite rubber

## Abstract

In this paper, the method of filling natural rubber with zeolite as filler was mainly studied in the following two aspects: firstly, experiments selected organic amine surface modifier to modify natural zeolite and used infrared spectroscopy to analyze the interaction between the modifier and zeolite, and secondly, studying the application of modified zeolite in natural rubber and using scanning electron microscopy to analyze the mechanism of action between zeolite and natural rubber. The test results show that octadecylamine in the modifier had the relatively best effect. Under the best conditions, the activation index of the modified product could reach 95% and above, and the contact angle could reach about 100°. When the filling amount was 30 phr, the octadecylamine modification had the most obvious effect on the performance of zeolite/natural rubber and the composite rubber had better mechanical properties. The corresponding tensile strength, tear strength, elongation at break and Shore hardness were 22.59 MPa, 28.52 MPa, 782.1% and 41, respectively, which were 45.74%, 19.28%, 7.95% and 7.89% higher than those of unmodified zeolite/natural rubber. As the first study of organic amine modified zeolite as a filler for filling natural rubber, this work provides a new way to improve the added value of natural zeolite.

## 1. Introduction

China is rich in natural zeolite resources, and its outstanding characteristics are large specific surface area, strong adsorption capacity, strong ion exchange capacity and low price [1]. Since natural zeolite has been used in industry, it has been mainly applied to three traditional fields based on its characteristics: (1) adsorption material [2,3], for industrial and environmental separation and purification, and for drying; (2) catalytic materials [4,5] for industrial catalysis in petroleum processing, the coal chemical industry and the fine chemical industry; (3) ion exchange materials [6,7], which are widely used in the detergent industry, mining and radioactive waste and waste liquid treatment. After decades of development, the application of natural zeolite has been effective in the traditional fields. However, as a non-metallic mineral with large reserves, it should find some new applications to increase its added value. In 1912, people found that carbon black had a reinforcing effect on rubber. Since then, carbon black has gradually become an indispensable raw material for the rubber industry [8]. The consumption of carbon black generally accounts for 40–50% of the consumption of rubber, ranking second in the consumption of raw materials in the rubber industry in the world. However, the production process of carbon black brings serious pollution to the environment and does great harm to the human body. On 27 October 2017, International cancer research units of the World Health Organization listed carbon black in the list of 2B carcinogens. In this case, it is urgent to find materials that can replace carbon black.

Natural zeolite is a kind of aluminosilicate mineral with two tetrahedral skeletons and pores. The tetrahedral center of the skeleton structure is occupied by silicon or aluminum atoms, and the four vertices are four oxygen atoms, respectively. The distance between Si-O ions is about 1.6 × 10^−10^ m, the distance between Al-O ions is about 1.75 × 10^−10^ m, and the distance between O-O ions is about 2.6 × 10^−10^ m. The Al^3+^ in the crystal structure replaces Si^4+^, which leads to the negative charge of the crystal. It needs to attract cations to achieve charge balance. The cations and anions are combined by electrostatic force, which is easy to exchange with cations in solution. This characteristic makes natural zeolite have strong ion exchange ability. Due to the openness of the crystal structure, zeolite contains many well-arranged, uniformly sized cavities and pores that are connected to each other and connected to the outside world, forming many wide, regular-shaped, and certain-sized cavities and channels connecting these cavities which constitute the unique structure of zeolite. The existence of a large number of cavities and voids means natural zeolite has a large specific surface area and strong adsorption capacity. These characteristics make it possible for natural zeolite to be used as a filler to fill natural rubber. At present, many non-metallic minerals, such as wollastonite, mica, diatomaceous earth, and talc, are used as fillers to fill rubber and other polymer materials, but research on the use of natural zeolite in this field is still low in China. Therefore, experiments on using modified natural zeolite as filler to fill polymer materials has certain research significance.

Organic amines are commonly used cationic surfactants. Their hydrophilic groups are positively charged. Natural zeolite surfaces are negatively charged in water. The hydrophilic groups of cationic surfactants can interact strongly with natural zeolite surfaces to form a tight adsorption layer, causing the hydrophobic nature of the zeolite surface. In this paper, zeolite minerals were used as raw materials to study zeolite-filled natural rubber in the following two aspects: firstly, organic amine surface modifier was selected to modify natural zeolite, and the interaction between the modifier and zeolite was analyzed by infrared spectroscopy and secondly, the application of modified zeolite in natural rubber was studied, and the mechanism of zeolite and natural rubber was analyzed by scanning electron microscope. Various zeolite fillers with different functions were prepared by exploring the effects of various modification factors on the performance of zeolite fillers. A new high quality zeolite filler for rubber was found by investigating the effect of zeolite fillers processed under different conditions on rubber properties.

## 2. Experimental Materials and Methods

### 2.1. Experimental Materials

The modified test raw material was a product obtained by mixing and grinding zeolite minerals from Liaoning Faku, with a particle size of D90 = 10.79 μm. Octadecylamine, analytically pure; Dodecyl tertiary amine, analytically pure, Beijing Jintong Letai Chemical Products Company (Beijing, China).

### 2.2. Experimental Method

#### 2.2.1. Surface Modification Test of Natural Zeolite

The surface modification of natural zeolite was divided into wet modification and dry modification [9]. Since the raw ore is wet stirred in a mill process, this experiment adopted the wet process. In the experiment, 22.5 g of ground ore with D_90_ = 10 μm was used as raw material, added to a 250 mL Erlenmeyer flask to prepare a modified slurry with a concentration of 15%, and then, the Erlenmeyer flask was put into a constant temperature water bath and the water temperature and speed of stirring were adjusted. After the natural zeolite powder was mixed with water, the modified agent was added when the modification temperature was reached and the modification time until the modification was completed was recorded. That is, a wet modified natural zeolite slurry was obtained. Then, the slurry was filtered by a vacuum filter and dried in an oven at a temperature within 80 °C. The dried filter cake was crushed by a pulverizer for use as a test sample.

#### 2.2.2. Application Testing of Zeolite Filled Rubber

The modified zeolite was blended with natural rubber according to different addition amounts, so that the natural zeolite particles were uniformly dispersed in the rubber matrix, and the rubber/natural zeolite composites were prepared. First, the natural rubber was mixed in the same direction into the wrapping drum in the open mill and mixing continued for 3 min. The rubber was wrapped in a drum for continuous rolling and refining, and 1 phr paraffin, 3 phr stearic acid, 1 phr antioxidant, 5 phr zinc oxide, 0.7 phr accelerator, 3 phr sulfur and 100 phr natural zeolite were successively added for mixing. In the process of mixing, a knife continuously cut the glue to make the mixing uniform, packing triangle bag eight times, thinned through ten times; and then, the drum spacing was adjusted to 2 mm and calendered for 1 min to produce the glue. The refined rubber was placed for 2 h and then molded on a flat vulcanizer at 144 °C for 25 min to form a rubber/natural zeolite composite test piece.

### 2.3. Characterization

The principle of the activation index was to use the hydrophobicity of the modified natural zeolite powder and determine the activation index by the mass of the natural zeolite powder sinking into the water in the total mass. The contact angle was measured by a contact angle measuring instrument. The material slice was obtained by a pressure mechanism, and, then, the slice was placed on a glass slide to ensure that the surface of the slice was level. The angle of contact measurement software was then launched to project the sheet surface onto the display screen and we then dropped a drop of water onto the solid sheet using a syringe on the tester, and the projection of water droplets on the solid surface was captured by the software shooting function. Finally, the contact angle of the material to be tested was measured by the software on the measuring instrument. The infrared spectrometer used in this study was a Perkin Elmer Spectrμm One FTIR Spectrometer (Shimadzu (Shanghai) Global Laboratory Consumables Co., Ltd., Shanghai, China) infrared spectrometer, with scanning resolution of 4 cm^−1^, and scanning of 128 times. Samples prepared by potassium bromide tableting were detected. The characterization of mechanical properties was mainly to indirectly characterize the quality of composites by measuring the mechanical properties of natural zeolite/natural rubber composites. The test indicators included: tensile strength, tear strength, tensile strength, elongation at break, permanent deformation at break, etc. The shore hardness test was to stack five samples together on a flat table, press the rubber surface with a durometer until the test index remained unchanged, and this was repeated three times to get the average value, and then, by using the scanning electron microscopy (SEM) (Carl Zeiss AG, Oberkochen, Germany) we observed the microscopic surface of the material.

## 3. Result 

### 3.1. Research on the Experimental Results of Zeolite Modification

It was necessary to modify the surface of the zeolite powder before filling the polymer to enhance its compatibility with the polymer. The hydrophilic group of organic amines are positively charged, and the surface of natural zeolite is negatively charged in water. The hydrophilic group of cationic surfactants form a strong interaction with zeolite, forming a tight adsorption layer, making the surface of zeolite hydrophobic [10]. Octadecylamine and Dodecyl tertiary amine are often used to modify the experiment under the condition of pH = 7, mainly to explore the three conditions of modification temperature, dosage and modification time.

#### 3.1.1. The Influence of Temperature and Dosage on Modification Effect

**Figure 1:**
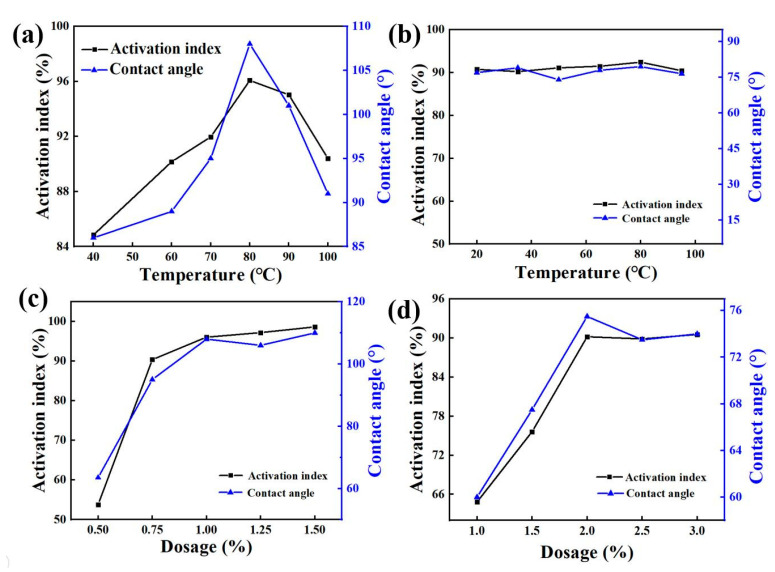
Effect of temperature on modification under the action of Octadecylamine (**a**) and Dodecyl tertiary amine (**b**); effect of dosage of Octadecylamine (**c**) and Dodecyl tertiary amine (**d**) on modification.

Figure 1 shows that when other conditions remained unchanged, the activation index and contact angle of zeolite-modified products first increased and then decreased with increase of the modification temperature. When the modification temperature was 40 °C, the activation index was 84.85% and the contact was 86.00°. When the modification temperature was between 40~80 °C, the activation index and contact angle increased with the increase of modification temperature. When the temperature reached 80 °C, the activation index was 96.07%, and the contact angle reached 108.00°. When the temperature increased, the activation index and contact angle of the modified product decreased. Then, the activation index and contact angle of the modified product became smaller when the temperature increased. When the temperature increased to 100 °C, the activation index became 90.40% and the contact was 91.00°, so the best modification temperature was 80 °C. The activation index and contact angle of modified zeolite products increased first, and then, tended to remain unchanged with the addition of dosages of stearylamine. When the dosage of the medicament was 0.5%, the activation index was 53.80%, and the contact angle was 63.50°. When the dosage of medicament was between 0.5~1.0%, the activation index and contact angle increased with the additional dosage. When the dosage of the medicament reached 1.0%, the index of activation was 96.84% and the contact angle reached 108.00°. If there was further increase in the dosage, the activation index and the contact angle tended to be stable. Therefore, the optimal dosage was 1.0%.

It can be seen from Figure 1 that when other conditions remained unchanged, the activation index and contact angle of the zeolite modified product did not change much with rise in the modification temperature. Therefore, 20 °C was selected as the optimal modification temperature condition, and the activation index was 90.76% and the contact angle was 77.00°. The activation index and contact angle of zeolite modified products increased first and then tended to be unchanged with increase of dosage of dodecyl tertiary amine. When the dosage of the medicament was 1.0%, the activation index was 64.85%, the contact angle was 60.00°, and when the dosage of the medicament was between 1.0~2.0%, the activation index and contact angle increased with the addition of dosage. When the dosage of the agent reached 2.0%, the activation index was 90.20%, the contact angle was 75.50°. Then, the activation index and contact angle became stable when the dosage continued increasing. Therefore, the optimal dosage was 2.0%.

#### 3.1.2. Effect of Modification Time on Modification

**Figure 2:**
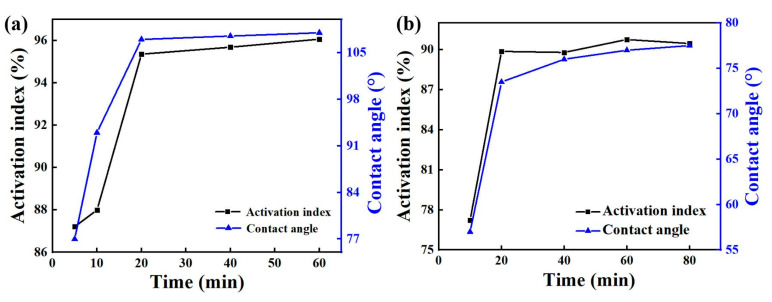
Effect of modification time of Octadecylamine (**a**) and Dodecyl tertiary amine (**b**) on modification.

The contact angle-activation index analysis of the two chemicals in Figure 2 shows that when other conditions remained unchanged, the activation index and contact angle modification time of the modified zeolite product first increased and then tended to remain unchanged. When the modification time was 5 min, the activation index was 87.21%, and the contact was 77.00°. For modification times between 5 min and 20 min, the activation index and contact angle increased with the modification time. When the modification time reached 20 min, the activation index was 95.36%, and the contact angle was 107.00°. With the increase of modification time, the activation index and contact angle tended to be stable. So, the optimal modification time of Octadecylamine was 20 min. When the modification time of Dodecyl tertiary amine was 10 min, the activation index was 77.23% and the contact was 57.00°. When the modification time was between 10 min and 20 min, the activation index and contact angle increased with the modification time. When it reached 20 min, the activation index was 89.87%, and the contact angle was 73.50°. With further increase of the modification time, the activation index and contact angle tended to be stable. Therefore, the optimal modification time of twelve tertiary amine was 20 min.

### 3.2. Effect of Filling Modified Zeolite on Rubber Properties

Natural rubber has good insulation, toughness, abrasion resistance, air-tightness in its water and gas barrier, plasticity resistance to tortuosity and strong elasticity [11]. However, in order to meet the needs of different applications, it must be improved by adding corresponding fillers. Analysis of aspects of the composition and crystal structure of natural zeolite, indicate that zeolite can be processed into fillers, and the use of zeolite as a non-metallic mineral filler can not only improve the performance of polymers to a certain extent, but can also reduce the content of the composite polymer, due to its light quality, so as to achieve the purpose of reducing costs and offering environmental protection.

When filling rubber, the surface modifier plays the role of connecting the rubber matrix and the zeolite, and also plays a different role in the rubber vulcanization process [12]. So, this experiment used the best modified octadecylamine to explore the influence of filling amount and modifier type on rubber performance in the control group experiments with filling amounts of 10 phr, 30 phr, 50 phr and 70 phr.

#### 3.2.1. The Effect of Zeolite Loading on the Tensile and Tear Strength of Rubber

**Figure 3:**
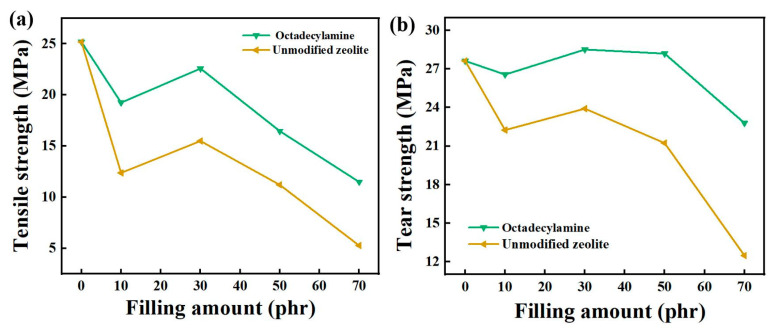
The effect of filling amount on tensile strength (**a**) and tear strength (**b**).

As shown in the Figure 3 below, the tensile strength and tear strength of the composite material first increased and then decreased with increase of fillers and the tensile strength reached its maximum when the filler content was 30 phr. When the filling content was 30 phr, the maximum was 22.59 MPa, which was 45.74% higher than that of unmodified zeolite at 15.50 MPa. When the content of zeolite was 10~70 phr, the tear strength of composite rubber increased first and then decreased with the increase of filler content. When the filler content was less than 30 phr, the tearing strength of the composite rubber gradually increased. When it was 30~50 phr, the tear strength was similar. When it was more than 50 phr, the tear strength began to decrease. The unmodified zeolite decreased more than the modified zeolite, which indicated that the modifier had a more obvious effect on the tear strength at a high filling amount. The maximum tear strength of unmodified zeolite was 23.91 MPa when the addition amount was 30 phr. Under the same filling amount, the maximum tear strength of composite rubber modified with octadecylamine reached 28.52 MPa, which was 19.28% higher than that of unmodified zeolite, and 3.26% higher than that of pure natural rubber (27.63 MPa). The modifier could improve the tear properties of zeolite/natural rubber, and the tear strength could be improved to better than that of natural rubber at a certain filling amount.

#### 3.2.2. The Effect of Zeolite Loading on Rubber Elongation at Break and Shore Hardness

**Figure 4:**
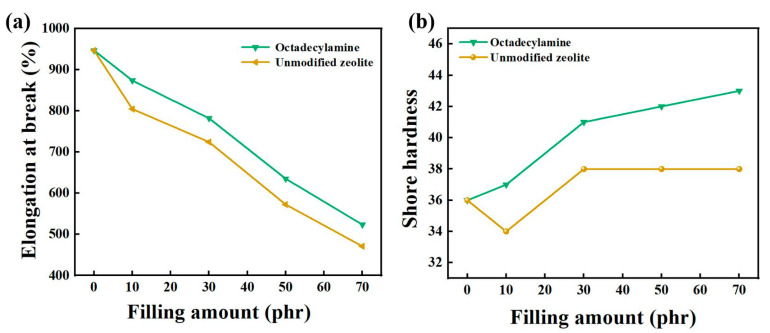
The effect of filling amount on elongation at break (**a**) and Shore hardness (**b**).

As shown in Figure 4, the elongation at break of composite rubber decreased with the increase of the zeolite filling fraction, and the unmodified zeolite increased first and then remained unchanged. Compared with the elongation at break of the composite rubber filled with unmodified zeolite, the modified treatment had an effect on the elongation at break. However, compared with the elongation at break of 947.10% of pure natural rubber, the elongation at break decreased after adding zeolite. When the addition amount was 30 phr, the elongation at break of the composite rubber filled with octadecylamine-modified zeolite was 782.10%, which was 7.95% higher than that of unmodified zeolite and 17.42% lower than that of natural rubber. The mechanical properties of the composite rubber obtained by octadecylamine-modified zeolite were relatively good. When the filling amount was 30 phr, its tensile strength, tear strength, elongation at break and Shore hardness were 22.59 MPa, 28.52 MPa, respectively, which were 45.74%, 19.28%, 7.95% and 7.89% higher than those of the composite rubber filled with unmodified zeolite. When the filling amount was greater than 30 phr, the tensile strength and tear strength began to decrease. The Shore hardness of the composite rubber increased with the addition of zeolite filler content. Therefore, octadecylamine was suitable for modifying natural zeolite, and the filling amount of suitable filler was determined to be 30 phr.

## 4. Analysis of the Modification Mechanism of Natural Zeolite and Study on the Mechanism of Filling Natural Rubber

### 4.1. Infrared Spectroscopy Analysis of Mechanism of Organic Amine Modified Zeolite

**Figure 5:**
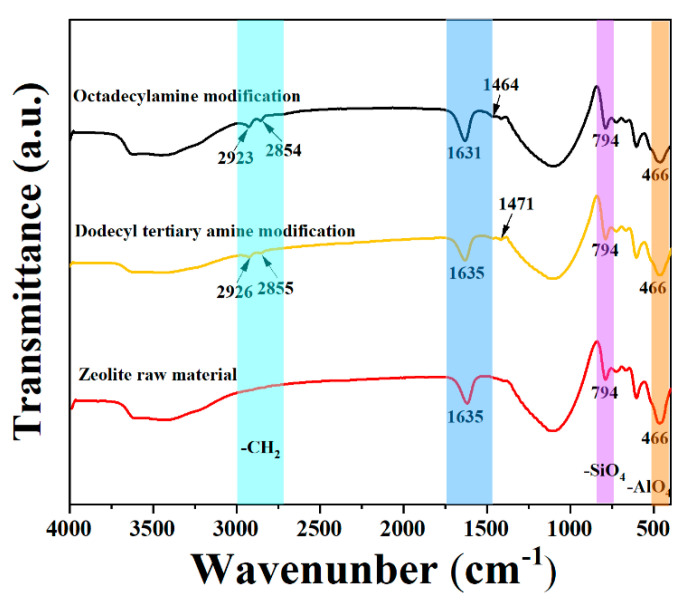
Comparison of infrared spectra of natural zeolite and organic amine modified products.

The infrared spectrum comparison between two amine-modified natural zeolite products and natural zeolite ore is shown in Figure 5. It can be seen that new absorption peaks appeared at 2927 cm^−1^, 2855 cm^−1^ and 1471 cm^−1^ for the products modified by Dodecyl tertiary amine. The products obtained after octadecylamine-modified zeolite also showed weak new absorption peaks at 2923 cm^−1^, 2854 cm^−1^ and 1463 cm^−1^, respectively. The vibration frequencies of the absorption peaks near 2925 cm^−1^ and 2855 cm^−1^ were the alkane CH_2_ antisymmetric and symmetric stretching vibration frequencies, respectively. The vibration frequencies of the left and right absorption peaks at 1465 cm^−1^ were the CH_2_ variable-angle vibration frequency, which further indicated the long-chain organic amines after the zeolite was modified by the medicament, wherein the medicament had been adsorbed on the surface of the zeolite, and the small peak of the new absorption peak indicated that the interaction between them was not strong. In addition, the absorption peak at 1200~400 cm^−1^ of the zeolite’s framework vibration did not change much, indicating that the modification did not destroy the zeolite structure lattice. The most basic structure of natural zeolite is silica (SiO_4_) tetrahedron and aluminum oxide (AlO_4_) tetrahedron, in which Al^3+^ will replace part of the Si^4+^ in the lattice structure, so that the surface of the zeolite is negatively charged, and the long-chain organic amine is soluble in water after it is positively charged, It can be inferred that amines and zeolite are electrostatically adsorbed.

### 4.2. Morphology Analysis of Compound Rubber after Adding Zeolite

#### 4.2.1. The Morphology Analysis of Compound Rubber with Different Additional Amounts of Unmodified Zeolite

**Figure 6:**
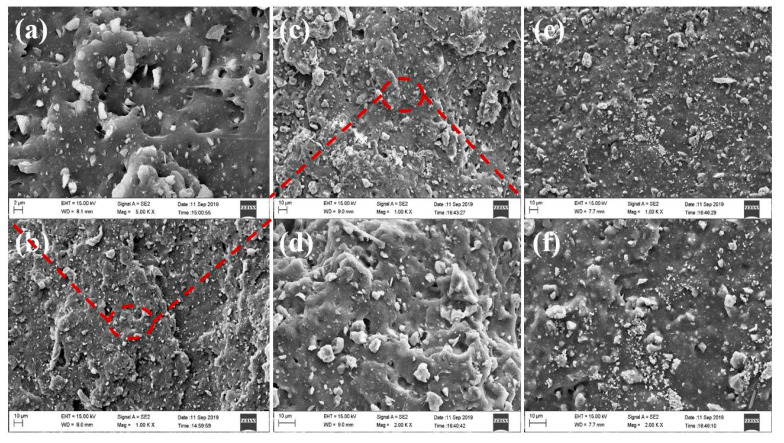
SEM pictures of unmodified zeolite/natural rubber under different loadings. Wherein (**a**,**b**) is an unmodified zeolite filled with 10 phr, (**c**,**d**) is filled with 30 phr, and (**e**,**f**) is filled with 70 phr.

Figure 6 shows the SEM images of natural rubber filled with different filling amounts of unmodified zeolite. The filling amounts were 10 phr, 30 phr and 70 phr, respectively. From Figure 6, it can be seen that the darker gray continuous phase in the figure is the natural rubber matrix, and the lighter off-white particles are the zeolite filler. It can be seen from 1000-fold SEM images that the agglomeration between filler particles became more and more obvious with increase of unmodified zeolite content. When the amount of unmodified zeolite was 10 phr and 30 phr, the dispersion was good, with less agglomeration, and the tear section showed uneven and irregular morphology [13]. When the addition amount was 70 phr, the agglomeration between particles was obvious, and the tearing section was smooth. This showed that the unmodified zeolite could interact better with natural rubber at low loadings, and withstand greater stress during tearing and fracture, resulting in irregular cross-sections. It could be seen from the 5000-fold SEM images that when the addition amount was 10 phr, the rubber matrix could cover the smaller particles in the filler. When the addition amount was 30 phr, a small amount of agglomeration of the filler occurred, but it could be clearly seen that there were filamentous long chains between the matrix. This might be due to the rubber polymer being adsorbed on the surface of the zeolite particles, so the tear strain would be stretched, indicating good interaction between the filler particles and the matrix. The agglomeration phenomenon was obvious when the addition amount was 70 phr, and the particles with smaller particle size would aggregate with each other due to the larger surface energy. In addition, due to the strong polarity of unmodified zeolite and the poor compatibility of natural rubber, when the composite rubber material was subjected to tear stress, the stress was concentrated on the interface between the filler and the matrix, the interface bonding force was weak, and the filler particles easily fell off from the matrix material, forming black holes in the SEM photograph of the tearing section; and it was observed that the interface between the filler particles and the matrix was clear, which also proved that the interaction between the unmodified zeolite and the rubber matrix was weak.

#### 4.2.2. Morphology Analysis of Compound Rubber with Modified Zeolite

**Figure 7:**
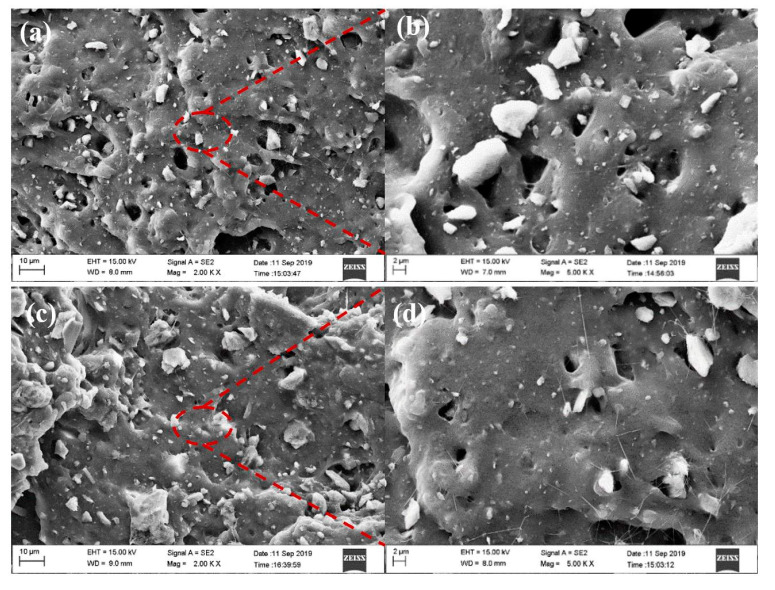
(**a**,**b**) SEM images of unmodified zeolite/natural rubber (**c**,**d**) Octadecylamine-modified zeolite/natural rubber.

It can be seen from Figure 7a,b that there are many black cavities in the tear section of the unmodified zeolite/natural rubber. This was caused by the zeolite particles being pulled out of the rubber matrix during the tearing and fracture processes, indicating that the interfacial bonding between unmodified zeolite and natural rubber was poor. Therefore, it could be seen that its mechanical properties were poor. From Figure 7c,d, it can be seen that the Octadecylamine-modified zeolite/natural rubber had fewer black cavities in the tearing section, the filler particles were more uniformly distributed, and the interface between the zeolite particles and the rubber matrix was blurred, and there seemed to be a layer of rubber matrix. It indicated that the zeolite modified by Octadecylamine could act well with the rubber, and the composite rubber could withstand greater external force when subjected to tearing strain [14,15], and its mechanical properties would be better.

In addition, EDS analysis was also carried out on the material, and the specific element composition is shown in Table 1.

## 5. Conclusions

In this paper, combined with the development and research status of natural zeolite, natural zeolite from Liaoning Faku was used as raw material to obtain modified zeolite by ultra-fine grinding and surface modification, and then it was filled into natural rubber to prepare zeolite/natural rubber composites. The effects of organic amine modifiers on the surface modification of natural zeolite under different modification conditions, and the effects of different filling amounts and modifier treatments on the properties of zeolite-filled natural rubber were investigated, and through infrared detection and scanning electron microscopy analyses, we obtained the following conclusions: (1) Among the two long-chain organic amine modifiers, Octadecylamine had the best effect. Under optimal conditions, the activation index of the modified products could reach 95% and above, and the contact angle could reach about 100°. (2) The mechanical properties of the composite rubber obtained by the Octadecylamine-modified zeolite filled with natural rubber were better, and with increase of natural zeolite, the mechanical properties of the composite rubber first increased and then decreased. At 30 phr, the mechanical properties were the best. At this time, the corresponding tear strength, tear strength, elongation at break and Shore hardness were 22.59 MPa, 28.52 MPa, 782.1% and 41, respectively, which were 45.74%, 19.28%, 7.95% and 7.89% higher than those of the composite rubber filled with unmodified zeolite, respectively. (3) Infrared spectrum analysis showed the physical adsorption caused by the hydroxyl association with silicone oil and natural zeolite. The electrostatic physical adsorption of organic amines and zeolite was due to the attraction of positive and negative charges. (4) Scanning electron microscopy analysis showed that with the increase of zeolite addition, the dispersion became worse and worse, and the agglomeration became more and more obvious. The interface between Octadecylamine- modified zeolite and the rubber matrix was blurred, whichindicated that there was an interaction between them.

## Figures and Tables

**Table 1:** Chemical compositions of the natural and modified zeolites by EDS (wt.%).

Chemical Elements	Natural Zeolite	Modified Zeolite
O	39.31	39.56
Si	43.2	41.3
Al	10.25	10.73
Na	1.25	0.67
Mg	0.46	1.23
K	1.35	0.46
Ca	2.32	0.37
N	0.23	3.45
Other	1.63	2.23

## Data Availability

The data are all original experimental data and are actually available.
